# Stage-Specific Alteration and Prognostic Relationship of Serum Fumarate Hydratase Autoantibodies in Gastric Cancer

**DOI:** 10.3390/ijms25105470

**Published:** 2024-05-17

**Authors:** Natsuko Sasajima, Makoto Sumazaki, Yoko Oshima, Masaaki Ito, Satoshi Yajima, Hirotaka Takizawa, Hao Wang, Shu-Yang Li, Bo-Shi Zhang, Yoichi Yoshida, Takaki Hiwasa, Hideaki Shimada

**Affiliations:** 1 Department of Gastroenterological Surgery, Toho University School of Medicine, Tokyo 143-8541, Japan; natsuko.kurihara@med.toho-u.ac.jp (N.S.); oshima@med.toho-u.ac.jp (Y.O.); tayajima@med.toho-u.ac.jp (S.Y.); 2 Department of Clinical Oncology, Toho University Graduate School of Medicine, Tokyo 143-8541, Japan; makoto.sumazaki@med.toho-u.ac.jp (M.S.); m.itoh@med.toho-u.ac.jp (M.I.); hiwasa_takaki@faculty.chiba-u.jp (T.H.); 3 Port Square Kashiwado Clinic, Kashiwado Memorial Foundation, Chiba 260-0025, Japan; QYW04410@nifty.com; 4 Department of Neurological Surgery, Chiba University Graduate School of Medicine, Chiba 260-8670, Japan; haowang@jnu.edu.cn (H.W.); lishuyang@zju.edu.cn (S.-Y.L.); dr.boshizhang@gmail.com (B.-S.Z.); y_yoichi0810@chiba-u.jp (Y.Y.)

**Keywords:** fumarate hydratase, autoantibody, gastric cancer, prognosis

## Abstract

The relationship between energy production and cancer is attracting attention. This study aimed to investigate the clinicopathological significance of fumarate hydratase (FH), a tricarboxylic acid cycle enzyme, in gastric cancer using autoantibodies as biomarkers. The study analyzed 116 patients who underwent gastric cancer surgery and 96 healthy controls. Preoperative serum FH autoantibody (s-FH-Ab) titers were analyzed using an immunosorbent assay with an amplified luminescent proximity homogeneous assay. Receiver operating characteristic analysis was used to determine the cutoff s-FH-Ab titer. Clinicopathological factors and prognosis were compared between the high and low s-FH-Ab groups. The s-FH-Ab levels were significantly higher in the gastric cancer group than in the control group (*p* = 0.01). Levels were elevated even in patients with stage I gastric cancer compared with healthy controls (*p* = 0.02). A low s-FH-Ab level was significantly associated with distant metastasis (*p* = 0.01), peritoneal dissemination (*p* < 0.05), and poor overall survival (*p* < 0.01). Multivariate analysis revealed that low s-FH-Ab levels were an independent risk factor for poor prognosis (*p* < 0.01). Therefore, s-FH-Ab levels may be a useful biomarker for early diagnosis and the prediction of prognosis in patients with gastric cancer.

## 1. Introduction

Gastric cancer is the fourth leading cause of cancer-related deaths worldwide [1]. Research into various biomarkers [2,3] and immune-related treatments [4,5] is ongoing to improve early diagnosis and prognosis.

Tumor cells are generally believed to consume high amounts of glucose to produce high energy levels [6]. In recent years, the relationship between cancer and glucose metabolism has attracted attention, and enzymes related to glycolysis and the tricarboxylic acid (TCA) cycle have been reported to be involved in cancer development and progression [7,8]. Metabolites or enzymes of the TCA cycle, such as fumarate hydratase (FH), isocitrate dehydrogenase, succinate dehydrogenase, and α-ketoglutarate dehydrogenase, are known to mutate or be deregulated in human cancers [9]. FH catalyzes malate production by the hydration of fumarate in the TCA cycle [10], and genetic mutations in FH have been reported to be associated with carcinogenesis [11]. Anupama et al. reported that low FH messenger ribonucleic acid levels were associated with lymph node metastasis, tumor histology, recurrence, and poor prognosis in lung adenocarcinoma [12].

Autoantibodies against tumor-associated antigens are used as markers to determine biological properties because they can be measured noninvasively and have a significant advantage in early detection compared with analysis using cancer cells [3]. Based on our experience of analyzing autoantibodies in cancer patients for over 20 years, we believe that glucose metabolism-related enzymes may function as antigens to induce autoantibodies in the blood of cancer patients. Because no study has analyzed autoantibodies against glycolytic enzymes or TCA cycle-related enzymes in solid tumors, this study aimed to investigate the clinicopathological and prognostic significance of serum FH antibodies (s-FH-Abs) in patients with gastric cancer.

## 2. Results

### 2.1. Comparison of s-FH-Ab Levels between Patients with Gastric Cancer and Healthy Donors and the Setting of Cutoff Values

**Figure 1:**
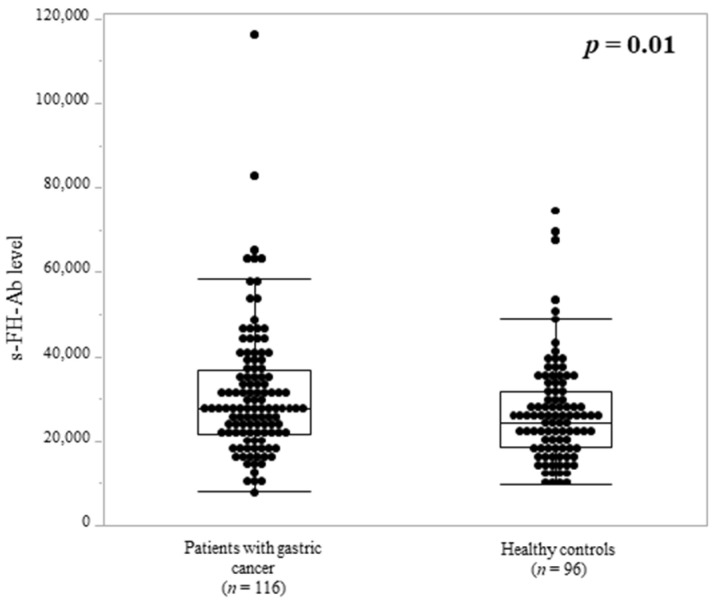
Comparison of serum fumarate hydratase antibodies (s-FH-Abs) between patients with gastric cancer and healthy controls. s-FH-Ab levels in the gastric cancer group and healthy control group were measured using amplified luminescent proximity homogenous assay (Alpha) with immunosorbent assay and are shown in scatter and box plots. The ordinate shows the Alpha photon counts representing s-FH-Ab levels. The box plots represent the 25th, 50th, and 75th percentiles. The upper and lower horizontal lines represent the 90th and 10th percentiles, respectively. *p* value was calculated by Mann–Whitney *U* test.

**Figure 2:**
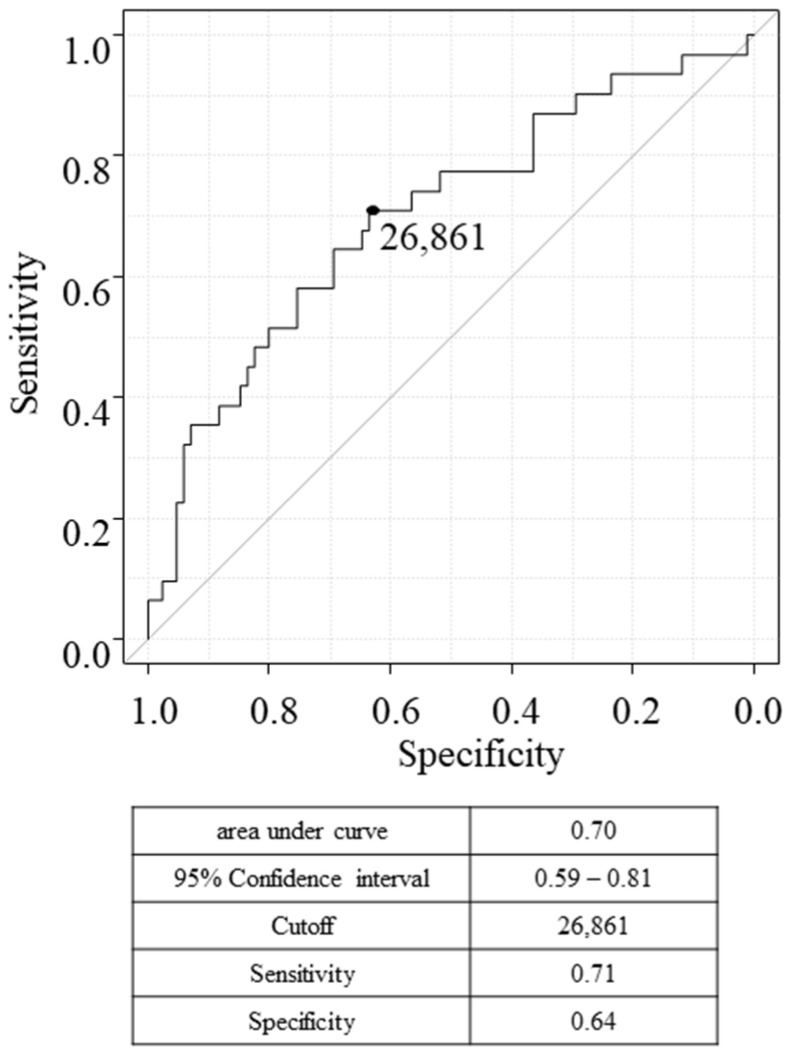
Receiver operating characteristic curve analysis of 116 surgically treated gastric cancer to predict overall survival. The numbers in the table represent the area under the curve, 95% confidence interval, cutoff level, specificity, and sensitivity. The black circle in the graph indicates the cutoff position at which the sum of sensitivity and specificity is maximized.

Recombinant FH protein was expressed in *Escherichia coli*, purified by affinity chromatography, and used as an antigen to measure serum antibody levels. s-FH-Ab levels were significantly higher in the gastric cancer group than in the healthy control group (*p* = 0.01, Figure 1). Receiver operating characteristic analysis, carried out to evaluate the ability of s-FH-Abs to indicate the presence of gastric cancer, showed an area under the curve of 0.70 and a cutoff value (Youden index) of 26,861 (Figure 2). The sensitivity and specificity were 0.71 and 0.64, respectively.

Patients with s-FH-Ab levels higher than the cutoff value were classified into the high s-FH-Ab group, whereas those with low s-FH-Ab levels belonged to the low s-FH-Ab group. 

### 2.2. Comparison of s-FH-Ab Levels by Stage

**Figure 3:**
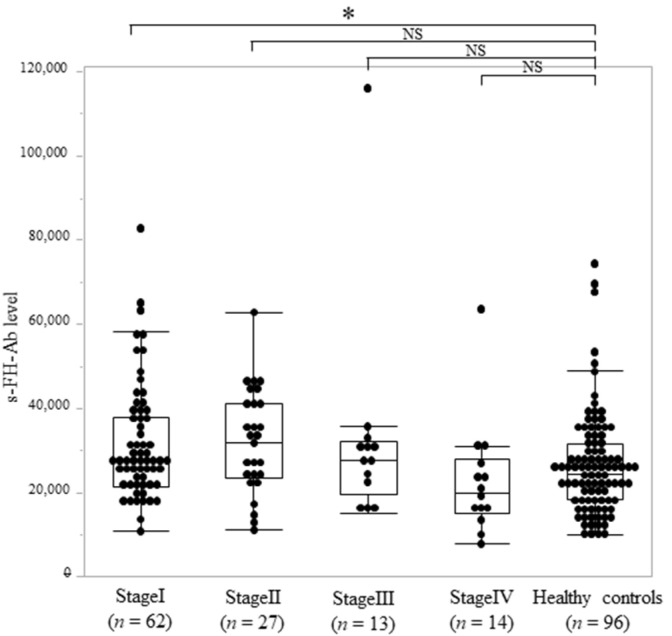
Comparison of s-FH-Ab levels between patients with gastric cancer and healthy controls by stage. The *p* values were calculated using the Kruskal–Wallis test (Mann–Whitney *U* test with Bonferroni correction). * *p* < 0.05. NS, not significant.

s-FH-Ab levels were compared among different stages. According to the Kruskal–Wallis test (Mann–Whitney *U* test with Bonferroni correction), s-FH-Ab levels by tumor stage were significantly different only in stage I in the gastric cancer group compared with those of the healthy control group (*p* = 0.02, Figure 3). However, as the stages progressed, the s-FH-Ab levels tended to decrease (Figure 3).

### 2.3. Correlation between Clinicopathological Factors and s-FH-Ab Levels

Table 1 shows a comparison of the clinicopathological factors between the high s-FH-Ab and low s-FH-Ab groups. No statistically significant differences in clinicopathological background, including age, sex, degree of tumor invasion, lymph node metastasis, distant metastasis, intraoperative peritoneal lavage cytology, stage, histological type, and tumor markers, were found between the two groups (Table 1). The low s-FH-Ab group showed significantly more distant metastases (*p* = 0.01) and more peritoneal metastases (*p* < 0.05) than the high s-FH-Ab group (Table 1). The low s-FH-Abs group showed a slight association with undifferentiated type (*p* = 0.10) and CA19-9 (*p* = 0.14); however, the differences were not statistically significant.

### 2.4. Logistic Regression Analysis of Clinicopathological Factors Associated with s-FH-Ab Levels

Table 2 shows the results of the logistic regression analysis using FH antibody titers as the dependent variable for items with *p* values < 0.01 in Table 1. No significant differences in nodal status, distant metastasis, peritoneal metastasis, and histology were observed. Therefore, no independent clinicopathological factors were associated with s-FH-Ab levels (Table 2).

### 2.5. Effect of High s-FH-Abs on Overall Survival

**Figure 4:**
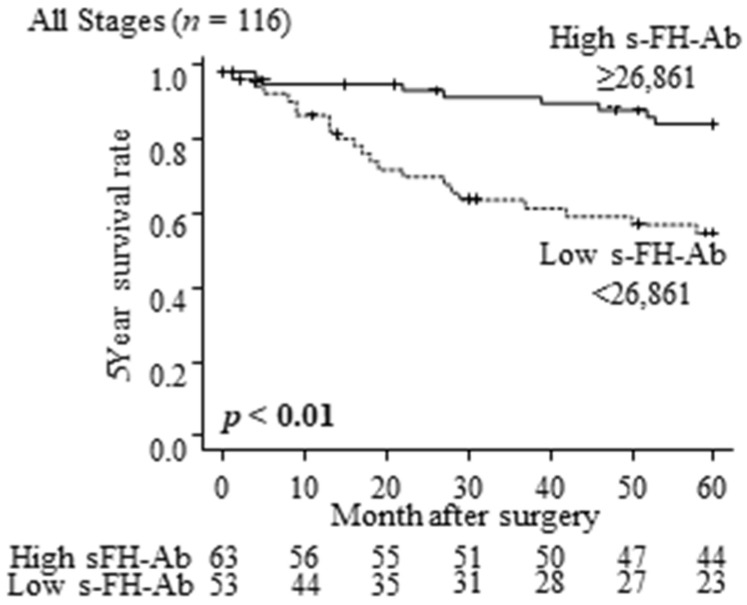
Comparison of overall survival according to s-FH-Ab levels at all stages using the log-rank test.

**Figure 5:**
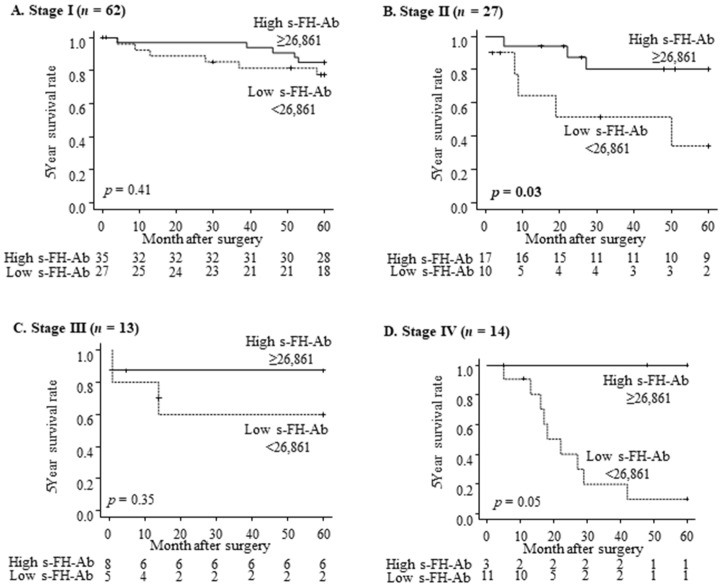
Comparison of overall survival according to the s-FH-Ab levels at each stage. Stages I (**A**), II (**B**), III (**C**), and IV (**D**). Evaluated by the log-rank test.

Figure 4 shows a comparison of overall survival between the high and low s-FH-Ab groups in patients with gastric cancer at all stages. The low s-FH-Ab group had significantly poorer overall survival than the high s-FH-Ab group (*p* < 0.01, Figure 4). Comparing the overall survival between the two groups at each stage, the low s-FH-Ab group had a significantly worse prognosis in stage II (*p* = 0.03, Figure 5B). Although similar tendencies were observed in stages I, III, and IV, the differences were not statistically significant (Figure 5A,C,D).

### 2.6. FH-mRNA Expression Levels at Each Stage and the Impact on Overall Survival

**Figure 6:**
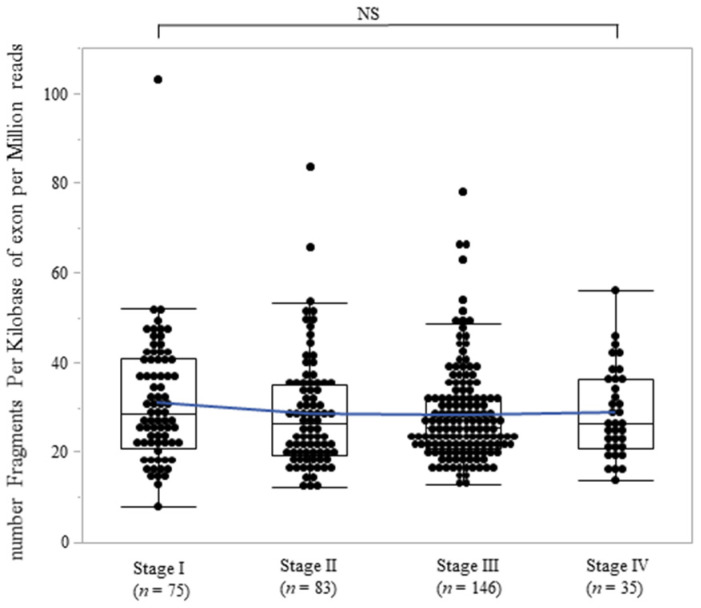
Comparison of FH-mRNA expression in stage I/II/III/IV gastric cancer tissues. RNA-seq data were generated by The Cancer Genome Atlas (TCGA). Normal distribution of FPKM (number of fragments per kilobase of exon per million reads) across the stages in gastric cancer tissues was visualized with box plots, shown as median and 25th and 75th percentiles. Outliners were all omitted. The blue line shows the mean FPMK of each stage. The Jonckheere–Terpstra test was utilized to test an ordered alternative hypothesis across the stages and FH-mRNA expressions. NS, not significant.

**Figure 7:**
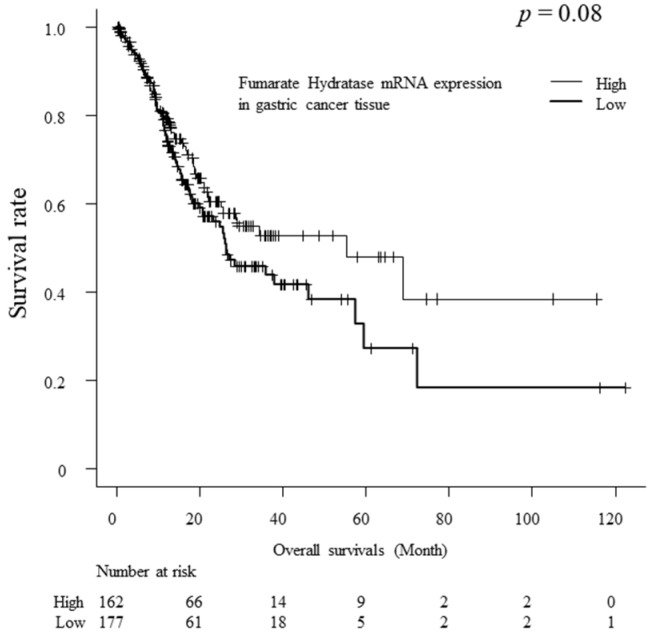
Comparison of overall survival according to FH-mRNA expression levels at all stages using the log-rank test. RNA-seq data and descriptions of each samples were generated by The Cancer Genome Atlas (TCGA).

The Cancer Genome Atlas (TCGA) program dataset was referred to in order to show the FH-mRNA expressions in gastric cancer tissues and their impact on survivals. Figure 6 shows the FH-mRNA expression levels at each stage. There were no statistically significant differences between each stage. Figure 7 shows a comparison of overall survival between the high and low FH-mRNA groups. The low FH-mRNA group had poorer overall survival than the high FH-mRNA group; however, the difference was not statistically significant (*p* = 0.08, Figure 7).

### 2.7. Univariate and Multivariate Analyses of the Prognostic Effect of Clinicopathological Factors

Our univariate analysis for overall survival showed that older age (≥65 years) (*p* = 0.02), advanced tumor invasiveness (*p* < 0.01), nodal metastasis (*p* = 0.02), distant metastasis (*p* < 0.01), and low s-FH-Ab levels (*p* < 0.01) are significant poor prognostic factors (Table 3). Based on the multivariate analysis, advanced tumor invasiveness (hazard ratio = 4.22, *p* < 0.01) and low s-FH-Ab levels (hazard ratio = 3.02, *p* < 0.01) are independently associated with poor prognosis (Table 3).

## 3. Discussion

This study investigated the clinicopathological and prognostic significance of preoperative s-FH-Abs in gastric cancer. s-FH-Ab levels were high in stage I gastric cancer but not in stages II–IV compared with those in the healthy control group (Figure 3). Low s-FH-Ab levels were significantly associated with distant metastasis (*p* = 0.01), peritoneal metastasis (*p* < 0.05) (Table 1), and poor overall survival (*p* < 0.01) (Table 3).

Tumor cells increase glucose uptake and utilization, promoting the TCA cycle [6], and are thought to increase TCA cycle enzymes and intermediates. Assuming that s-FH-Ab reflects the amount of FH proteins, the emergence of s-FH-Abs in the early stages of gastric cancer may be caused by the excessive oxygen demand that occurs with tumor growth/spread. As the disease progresses, FH production may be reduced as additional oxygen demand occurs, inducing an anaerobic environment in which the TCA cycle is relatively inhibited. Alternatively, host immunity may act as a tumor suppressor mechanism during the early stages of carcinogenesis. This host immunity can be lost as the cancer progresses.

The poor prognosis of the low s-FH-Ab group was possibly related to FH gene mutations and/or FH inactivation, the subsequent suspension of the TCA cycle, and the subsequent accumulation of the substrate fumarate. Fumarate accumulation was found to have cytotoxic effects, in addition to intracellular protein modifications and associated compensatory metabolic changes [13]. The accumulated fumarate permeates multiple compartments, including the mitochondria, cytoplasm, and nucleus, causing changes in various signaling cascades [9,14,15]. Fumarate accumulation also leads to succinate accumulation, and their accumulation inhibits pyruvate dehydrogenase kinase 1 [16,17]. Pyruvate dehydrogenase kinase 1 stabilizes hypoxia-inducible factor 1-α by inhibiting prolyl hydroxylate [16,18]. Impaired mitochondrial function through the disruption of the TCA cycle and the inhibition of this prolyl hydroxylase result in a shift to aerobic glycolysis involving lactate production and the pentose phosphate pathway [19,20]. These compensatory metabolic changes allow cancer cells to continue producing energy even when FH mutated or is inactivated. Furthermore, the stabilization of hypoxia-inducible factor 1-α activates hypoxia-inducible factor-related signaling cascades, promotes angiogenesis and tumor growth, and contributes to cancer progression and malignant transformation [16,21]. Furthermore, increased nuclear fumarate levels may cause the dysfunction of enzymes that regulate chemical changes in deoxyribonucleic acids and histones, such as ten-eleven translocated proteins [22] and lysine demethylases [23]. For example, the inhibition of ten-eleven translocation-dependent deoxyribonucleic acid demethylation suppresses microRNA 200, an anti-metastatic microRNA family [24], triggering an epithelial-to-mesenchymal transition that promotes metastatic dissemination [25,26]. Low FH levels are thought to influence cancer cell progression through these multiple step-by-step mechanisms [11,27], and FH plays an important role in energy acquisition and cancer progression.

This study showed that distant metastasis, peritoneal metastasis, and overall survival were higher in the low s-FH-Ab group compared with the high s-FH-Ab group. This consistently promotes malignant transformation and the metastasis of cancer cells brought about by the aforementioned low FH levels. 

Cases with high autoantibody titers have high expression levels of target proteins [28]. To the best of our knowledge, this is the first study to confirm the presence of auto-antibodies against glucose metabolism-related enzymes, suggesting the potential of autoantibodies as surrogate markers

Based on Human Protein Atlas data [29], the overall survivals were compared between the high mRNA expression group and low mRNA group. Although the high mRNA expression group showed relatively better survival than the low mRNA expression group, the difference was not statistically significant. Therefore, s-FH-Ab analysis may be a better biomarker than FH mRNA analysis for the impact of survival.

One of the most important findings in this study is that FH autoantibody levels in stage I are significantly higher than in healthy subjects, but from the perspective of false positive rates, it is difficult to screen for early cancer using FH autoantibodies alone. It is necessary to establish an effective early gastric cancer diagnosis method by combining multiple biomarkers or other diagnostic methods.

This study has several limitations. First, the correlation between enzyme activity and autoantibodies was not assessed. Correlations among existing FH autoantibodies, protein expression, and FH enzyme activity are working hypotheses to be clarified in the future. Second, because s-FH-Ab levels were not measured after surgery, the perioperative changing patterns of s-FH-Ab levels are unclear. Third, because the study cohort was a test cohort, a large multi-institutional cohort is required for evaluation. Fourth, since other TCA cycle-related enzymes are potential targets of autoantibodies, further investigations aiming to set up new system to analyze autoantibodies against those enzymes are required. In the future, the response of s-FH-Ab levels to treatment and how autoantibodies change during cancer recurrence must be analyzed.

## 4. Materials and Methods

### 4.1. Ethical Approval and Informed Consent

The study was conducted following the guidelines of the Declaration of Helsinki. The collection of serum samples was approved by the Ethics Committee of Faculty of Medicine, Toho University (Nos. A18103_A17052_A16035_A16001_26095_25024_24038_22047, 25131_23005), Toho University Omori Medical Center (No. 26-255), Chiba University Graduate School of Medicine (No. 2018-320), and Port Square Kashiwado Clinic, Kashiwado Memorial Foundation (No. 2012-001). Written informed consent was obtained from all patients. The retrospective analysis of patients’ medical records was approved by the Ethics Committee of Faculty of Medicine, Toho University (No. A22038_A21089_A19030), and Toho University Omori Medical Center (No. M22211). The potential participants were given the opportunity to decline to be further enrolled in the study (opt out).

### 4.2. Participants and Sera

The participants included 116 patients with gastric cancer who underwent radical surgery at Toho University Omori Hospital between 2008 and 2013. They were followed up until death or the end of 2022. There were 79 men and 37 women, and their average age was 68 (range, 39–92) years. The control group consisted of 96 healthy individuals who visited Port Square Kashiwado Clinic. This group included 51 men and 45 women, and their mean age was 58 (range: 50–76) years. The pathological stages using resected specimens (14th edition of gastric cancer handling regulations [30]) were stages I (*n* = 62), II (*n* = 27), III (*n* = 13), and IV (*n* = 14).

### 4.3. Purification of Recombinant Proteins

ECOS^TM^ competent *Escherichia coli* BL21-109 cells (Nippon Gene) were transformed with the eukaryotic expression plasmid, pGEX-4T-1 or pGEX-4T-1-FH, and then cultured for 3 h in 200 mL of Luria broth containing 0.1 mM isopropyl β-D-thiogalactopyranoside (IPTG; Wako Pure Chemicals, Osaka, Japan) [31]. The cells were then harvested, washed with phosphate-buffered saline, and lysed by sonication in BugBuster Protein Extraction Reagent (Novagen, San Diego, CA, USA). Lysates were centrifuged at 15,000× *g* for 10 min at 4 °C, and glutathione S-transferase (GST) and GST-fused FH proteins were purified using affinity chromatography with glutathione–Sepharose columns (Cytiva, Pittsburgh, PA, USA) as previously described [32].

### 4.4. Measurement of s-FH-Ab Levels and Conventional Serum Markers

Serum samples were collected before treatment, centrifuged at 3000× *g* for 10 min, and stored at −80 °C until use. s-FH-Ab levels were measured using an amplified luminescence proximity homogeneous assay-linked immunosorbent assay (AlphaLISA) for FH. AlphaLISA was performed using 384-well microtiter plates (white opaque OptiPlate™, Revvity, Waltham, MA, USA) containing 2.5 μL of 1/100-diluted sera and 2.5 μL of GST or GST fusion proteins (10 μg/mL) in AlphaLISA buffer (25 mM HEPES, pH 7.4, 0.1% casein, 0.5% Triton X-100, 1 mg/mL dextran-500, and 0.05% Proclin-300) according to the manufacturer’s instructions (Revvity, “http://www.perkinelmer.com/lab-solutions/resources/docs/GDE_ELISA-to-AlphaLISA.pdf (accessed on 7 May 2024)”). The reaction mixture was incubated at room temperature for 6–8 h. Then, anti-human IgG-conjugated acceptor beads (2.5 μL of 40 μg/mL) and glutathione-conjugated donor beads (2.5 μL of 40 μg/mL) were added and incubated further for 7–21 days at room temperature in the dark. The chemical emission was read on an EnSpire Alpha microplate reader (Revvity) as described previously [33]. Specific reactions were calculated by subtracting the Alpha values of the GST control from the values of GST fusion proteins.

CEA levels were measured using a CEA-2 enzyme immune assay kit (Elecsys CEAII; Roche Diagnostics K.K., Tokyo, Japan) according to the manufacturer’s instructions. The cutoff value was 5.0 ng/mL. CA19-9 levels were measured using a CA19-9 enzyme immune assay kit (Elecsys CA19-9; Roche Diagnostics K.K., Tokyo, Japan). The cutoff value was 37 U/mL [34].

### 4.5. The Cancer Genome Atlas Program (TCGA) Data-Based Analysis in Gastric Cancer

TCGA data were obtained from Protein Atlas Ver 23.0. [29]. Patient descriptions (stage and prognosis) of gastric cancer and the RNA-seq of their tumor tissues in the TCGA dataset all referred to the Pathology link in the Human Protein Atlas [35].

### 4.6. Comparison of Overall Survivals between High FH mRNA Expression Group and Low FH mRNA Expression Group

mRNA expression data were extracted from the Human Protein Atlas [29]. The best expression cutoff refers the optimal cut off value that yields maximal difference with regard to survival between the two groups at the lowest log-rank *p* value. Best expression cutoff was selected based on survival analysis.

### 4.7. Statistical Analysis

The patients were classified into the high and low s-FH-Ab groups, and analyses were performed subsequently. We utilized the Mann–Whitney *U* test, Fisher’s exact test, and the Kruskal–Wallis test (Mann–Whitney *U* test with Bonferroni correction) to determine significant differences between two groups and between three or more groups, respectively. Survival-related clinicopathological parameters were evaluated by univariate analysis using the log-rank test based on Kaplan–Meier survival curves. Multivariate analysis was performed using the Cox proportional hazards model. The Jonckheere–Terpstra test was utilized to test an ordered alternative hypothesis. Statistical analysis was performed using R (The R Foundation for Statistical Computing; version 2.13.0), graphical user interface EZR (Jichi Medical University Saitama Medical Center; Saitama, Japan) [36], or JMP Pro v17.0.0 (SAS Institute, Inc., Cary, NC, USA); *p* values < 0.05 were considered statistically significant.

## 5. Conclusions

In this study, s-FH-Abs were overexpressed in the early stages of gastric cancer and gradually decreased with cancer progression. A low s-FH-Ab level was an independent risk factor for poor prognosis and was associated with the malignant progression potential of gastric cancer. Therefore, s-FH-Abs may be useful for early diagnosis and for predicting overall survival.

## Figures and Tables

**Table 1:** Comparison of the frequency of high s-FH-Ab titers according to the clinicopathological variables.

Variables	High s-FH-Ab Group ≥ 26,861 *n* = 63 (%)	Low s-FH-Ab Group < 26,861 *n* = 53 (%)	*p* Value *
Age			>0.99
<65	26 (41.3)	21 (39.6)	
≥65	37 (58.7)	32 (60.4)	
Sex			0.24
Female	17 (27.0)	20 (37.7)	
Male	46 (73.0)	33 (62.3)	
Tumor depth			0.26
T1	30 (47.6)	19 (35.8)	
T2/T3/T4	33 (52.4)	34 (64.2)	
Nodal status			0.05
Negative	45 (71.4)	28 (52.8)	
Positive	18 (28.6)	25 (47.2)	
Distant metastasis			0.01
Negative	60 (95.2)	42 (79.2)	
Positive	3 (4.8)	11 (20.8)	
Peritoneal metastasis			<0.05
Negative	62 (98.4)	47 (88.7)	
Positive	1 (1.6)	6 (11.3)	
Intraoperative peritoneal lavage cytology			0.57
CY0	57 (90.5)	46 (86.8)	
CY1/X	6 (9.5)	7 (13.2)	
Stage			0.71
I	35 (55.6)	27 (50.9)	
II/III/IV	28 (44.4)	26 (49.1)	
Histology			0.10
Differentiated	34 (54.0)	20 (37.7)	
Undifferentiated	29 (46.0)	33 (62.3)	
CEA (ng/mL)			>0.99
<5.0	52 (82.5)	43 (81.1)	
≥5.0	11 (17.5)	10 (18.9)	
CA19-9 (U/mL)			0.14
<37	61 (96.8)	47 (88.7)	
≥37	2 (3.2)	6 (11.3)	

* Fisher’s exact probability test. Significant correlations (*p* < 0.05) are in boldface.

**Table 2:** Logistic regression analysis of clinicopathological factors associated with s-FH-Ab levels.

Variables	Odds Ratio	95% Confidence Interval	*p* Value *
Nodal status			
Negative/Positive	1.63	0.25–3.96	0.28
Distant metastasis			
Negative/Positive	2.29	0.67–14.10	0.37
Peritoneal metastasis			
Negative/Positive	2.06	0.37–30.50	0.60
Histology			
Differentiated/Poor	1.66	0.75–3.65	0.21

* Logistic regression analysis.

**Table 3:** Univariate and multivariate analyses of clinicopathological factors and serum biomarkers to predict overall survival.

Variables	Univariate *p* Value *	Multivariate Analysis		
		Hazards Ratio	95% Confidence Interval	*p* Value **
Age			0.83–4.79	0.12
≥65	**0.02**	2.00		
<65				
Sex				
Male	0.12			
Female				
Tumor depth			1.42–12.56	**<0.01**
T2/T3/T4	**<0.01**	4.22		
T1				
Nodal status			0.29–2.08	0.61
Positive	**0.02**	0.77		
Negative				
Distant metastasis			0.84–6.54	0.10
Positive	**<0.01**	2.35		
Negative				
Histology				
Poor	0.60			
Differentiated				
CEA (ng/mL)				
≥5.0	0.41			
<5.0				
CA19-9 (U/mL)				
≥37	0.10			
<37				
s-FH-Ab			1.36–6.71	**<0.01**
<26,861	**<0.01**	3.02		
≥26,861				

* Log-rank test analysis; ** Cox proportional hazards regression analysis. Significant correlations (*p* < 0.05) are in boldface.

## Data Availability

The datasets used and/or analyzed in the present study are available from the corresponding author on reasonable request.
